# Supplementary material for: Optimization of Transposon Mutagenesis Methods in Pseudomonas antarctica
Source: Microorganisms. 2023 Jan 1;11(1):118. doi: 10.3390/microorganisms11010118 (PMC9864612; doi:10.3390/microorganisms11010118)
Supplement: Supplementary file 1 [file microorganisms-11-00118-s001.zip › microorganisms-2076778-supplementary.pdf]

[Supplemental Information]

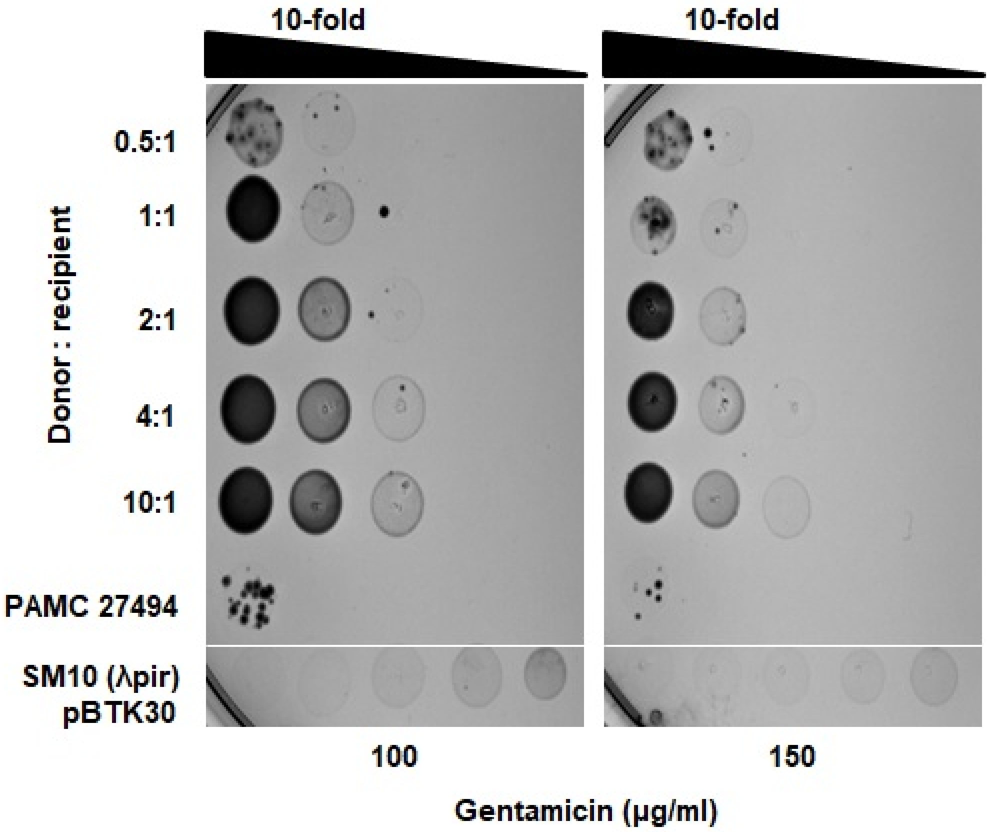

Supplement Figure S1. Conjugation efficiency at low gentamicin concentrations

The conjugation efficiency at low gentamicin concentrations in the selection plate was tested.

CGATAAGATAGAAACCGTGGATCTTATGAGCCGCCTGTTAAGTGCACGAGC
ATCTCACCATTTTTGCTTGCGACGCTGAGACTGGTTCTGTTGTCTTCTTCC
ACGCCTGGGGGGGCGACCTTCGCCGCGTCGGTGTTTCCTAGCAAAATGG
GAAATTTTGTTTGGGGGAAGGACCGAAAATGACTGATAGAGCCCGTCAGG
ACATACCCTGGGTCTGACACAGAATGCAGCACAAAATTCGCCGCAATGGC
ACAATTGCCCCGCTGTGCCCTCCCCCAAACAGGATTTCCCATGCTGCAAC
TGAACACCGACGCGCTGATGGCCACCCCGTGCGACGACGAAGAAGACAA
CATGGCCATGCTCTGCTGCCACGGTAAAAACGGCGAGATGTTCATGCTCA
CCCGTTACCCGGACGAAGATGAAGTCGAACTGACTTGGGATTACGAGCCG
TCGACCTGCAGGCATGCACA

Supplement Figure S2. Sanger sequencing result of inverse-PCR products

Transposon insertion site was determined by inverse PCR and subsequent Sanger sequencing. The genomic locus in *P. antarctica* containing transposon was highlighted in yellow and transposon region was not highlighted.
